# Analysis of risk factors of rapid thyroidal radioiodine-131 turnover in Graves’ disease patients

**DOI:** 10.1038/s41598-017-08475-z

**Published:** 2017-08-15

**Authors:** Ruiguo Zhang, Jian Tan, Renfei Wang, Guizhi Zhang, Qiang Jia, Zhaowei Meng, Yueqian Zhang

**Affiliations:** Department of Nuclear Medicine, Tianjin Medical University General Hospital, Tianjin, China

## Abstract

Rapid iodine-131(^131^I) turnover in the thyroid gland is an important feature of Graves’ disease (GD) and also a strong predictor of radioiodine therapy failure. The aim of this study was to explore the predictors of rapid ^131^I turnover. The clinical data on 2543 patients were retrospectively reviewed. Patients were divided into 2 groups depending on present or absent with rapid ^131^I turnover defined as a 4-hour to 24-hour ^131^I uptake ratio of ≥1. Overall, 590 cases (23.2%) had a rapid ^131^I turnover. In the univariate analysis, gender, age, FT_3_/FT_4_ concentration, disease duration, with or without antithyroid drugs (ATD), time of ATD, thyroid weight and thyroid textures displayed significant differences. Cutoff values of age, FT_3_ and thyroid weight to predict rapid ^131^I turnover were 38 years, 35 pmol/l and 56 g by receiver operating characteristic curves. Binary logistic regression analysis further revealed higher probability of rapid ^131^I turnover in patients with thyroid weight ≥56 g (odds ratio [OR]:3.7, 95% confidence interval [CI]: 3.032–4.559), age <38 years (OR:2.3, 95%CI: 1.906–2.856), FT_3_ concentration ≥35 pmol/l (OR:7.6, 95%CI: 5.857–8.563) and females (OR:2.2, 95%CI: 1.757–2.791). In conclusion, larger goiters, younger age, higher FT_3_ concentration and females are independently associated with rapid ^131^I turnover in GD patients.

## Introduction

Graves’ disease (GD) is the commonest cause of hyperthyroidism and accounted for nearly 90% cases. As an autoimmune disorder, women are more susceptible to hyperthyroidism than men^1^. Radioiodine-131 (^131^I), which has been used for approximately 70 years for the treatment of GD, was proven to be efficient and safe both as a primary therapy and secondary therapy when thyrotoxicosis can not be controlled by antithyroid drugs (ATD)^2–4^.

Radioactive iodine uptake (RAIU) is a useful tool which can be used to differentiate hyperthyroidism from thyroiditis, calculate the ^131^I dose for treatment of GD and predict therapeutic outcome^5, 6^. The dose of ^131^I to be administered could be fixed even if neither thyroid weight nor thyroid uptake is known and also adjusted using complex dosage formula based on gland size, the 131I concentration of per gram thyroid tissue as well as the residence time of ^131^I in the thyroid gland^7–10^.

At some institutions, traditionally, only 24-hour RAIU was measured for calculation of therapeutic doses of ^131^I^11^. However, it was impossible to calculate the residence time of thyroidal ^131^I by using a single measurement. It was reported that 4- to 24-hour uptake ratio could be used as an index of rapid iodine-131 turnover and a surrogate parameter for effective half life in hyperthyroidism^12^. Previous studies showed that about 12–32% GD patients had shortened residence time of ^131^I in the thyroid gland due to the rapid ^131^I turnover^12–14^. Additionally, several investigators reported that rapid ^131^I turnover was a strong predictor of radioiodine therapy (RIT) failure^12, 14, 15^, which increased the whole-body radiation dose secondary to the additional release of protein-bound ^131^I into the vascular system^16^. However, to our knowledge, there is lack of study to reveal the predictive factors of rapid ^131^I turnover in patients with GD.

In the present study, our aim was to explore the potential factors that could predict rapid thyroidal ^131^I turnover in patients with GD by retrospective review of their data.

## Results

### Patients and first-dose RIT failure

Baseline and pre-RIT patient clinical characteristics of the 2543 patients studied are listed in Table 1.

**Table 1:** Baseline and pre-RIT patient clinical characteristics (n = 2543).

Characteristic	
Gender	
Male	793(31.2%)
Female	1750(68.8%)
Age(years)	
Mean ± SD	40.8 ± 13.6
Median (range)	40.0(10–80)
FT_3_(pmol/l)	
Mean ± SD	34.2 ± 11.2
Median (range)	33.6(6.6–50)
FT_4_(pmol/l)	
Mean ± SD	79.3 ± 19.6
Median (range)	83.4(28.0–100)
Disease duration (mons)	
Mean ± SD	31.8 ± 42.8
Median (range)	12.0(0.2–360)
ATD	
With	1742(68.5%)
Without	801(31.5%)
Time of ATD(mons)	
Mean ± SD	18.3 ± 31.6
Median (range)	3.0(0–240)
4 h thyroid ^131^I uptake(%)	
Mean ± SD	41.3 ± 20.6
Median (range)	40(7–92)
24 h thyroid ^131^I uptake(%)	
Mean ± SD	75.6 ± 19.5
Median (range)	76(32–96)
Thyroid weight(g)	
Mean ± SD	58.6 ± 27.0
Median (range)	52.0(15–212)
Complications	
With	662(26.0%)
Without	1881(74.0%)
Thyroid nodules	
With	36414.3%)
Without	2179(85.7%)
Thyroid texture	
Soft	1275(50.1%)
Moderate	943(37.1%)
Stiff	325(12.8%)

Data are presented as count (percentage) or mean ± SD and median (range). RAIU = radioiodine uptake, FT_3_ = free triiodothyronine, FT_4_ = free thyroxine, SD = standard deviation, ATD = antithyroid drugs.

Overall, 195 cases (7.7%) remained hyperthyroid after first-dose RIT (first-dose failure), and therefore, were given repeated RIT. Of them, 177 cases (7.0%) received second, 15 cases third and 3 subjects fourth ^131^I. Additionally, 590 patients (23.2%) had a rapid ^131^I turnover.

### Comparison of patient characteristics between groups

Demographic and clinical characteristics of the 2 group patients studied are displayed in Table 2. We found subjects with rapid ^131^I turnover had a higher first-dose failure rate (12.0% vs. 6.3%, OR = 2.018, 95%CI: 1.484–2.744, *P* < 0.0001). When we compared the categorical variables between groups using the chi square test, we found no statistically significant association in the complications (*P* = 0.782) and thyroid nodules (*P* = 0.643). However, there was significant difference in the gender composition (*P* < 0.0001), thyroid textures (*P* = 0.004) and with or without ATD (*P* < 0.0001).

**Table 2:** Comparison of patient characteristics between the 2 groups.

Characteristic	Rapid ^131^I turnover		*P*
	Present (n = 590)	Absent (n = 1953)	
Gender			
Male	126(21.4%)	667(34.2%)	<0.0001*
Female	464(78.6%)	1286(65.8%)	
Age(years)	33.0(11–78)	42.0 (10–80)	<0.0001**
FT_3_(pmol/l)	38.2(11.6–50)	31.3(6.6–50)	<0.0001**
FT_4_(pmol/l)	86.5(32.2–100)	78.9(28.0–100)	0.0010**
Disease duration (mons)	12.5(0.3–264)	12.0(0.2–360)	0.1090
ATD			
With	442(74.9%)	1300(66.6%)	<0.0001*
Without	148(25.1%)	653(33.4%)	
Time of ATD	6.0(0–240)	2.0 (0–216)	0.0010**
4 h thyroid ^131^I uptake(%)	42(7–89)	40(9–92)	0.2060
24 h thyroid ^131^I uptake(%)	75(35–96)	77(32–95)	0.5340
Thyroid weight(g)	68.0(20–212)	48.5(15–212)	<0.0001**
Complications			
With	151(25.60%)	511(26.2%)	0.7820
Without	439(74.4%)	1442(73.8%)	
Thyroid nodules			
With	81(13.7%)	283(14.5%)	0.6434
Without	509(86.3%)	1670(85.5%)	
Thyroid textures			
Soft	266(45.1%)	1009 (51.7%)	0.0040*
Moderate	236(40.0%)	707(36.2%)	
Stiff	88(14.9%)	237(12.1%)	
First-dose ^131^I failure			
With	71(12.0%)	124(6.3%)	<0.0001*
Without	519(88.0%)	1829(93.7%)	

Data are presented as count (percentage) or median (range). *P value < 0.01 using chi square test. **P value < 0.01 using Mann–Whitney U test. FT_3_ = free triiodothyronine, FT_4_ = free thyroxine, SD = standard deviation, ATD = antithyroid drugs.

Similarly, we found no statistically significant difference in the disease duration (*P* = 0.109), 4 h/24 h thyroid ^131^I uptake (*P* = 0.206 and 0.534, respectively) when comparing the continuous variables in the 2 groups using the Mann-Whitney U test. However, we found younger patients, cases with higher FT_3_/FT_4_ concentration and heavier thyroid weight, and those with longer time of ATD use more likely had rapid ^131^I turnover (all *P* < 0.01).

### Diagnostic values of age, FT_3_ and thyroid weight for rapid ^131^I turnover

**Figure 1:**
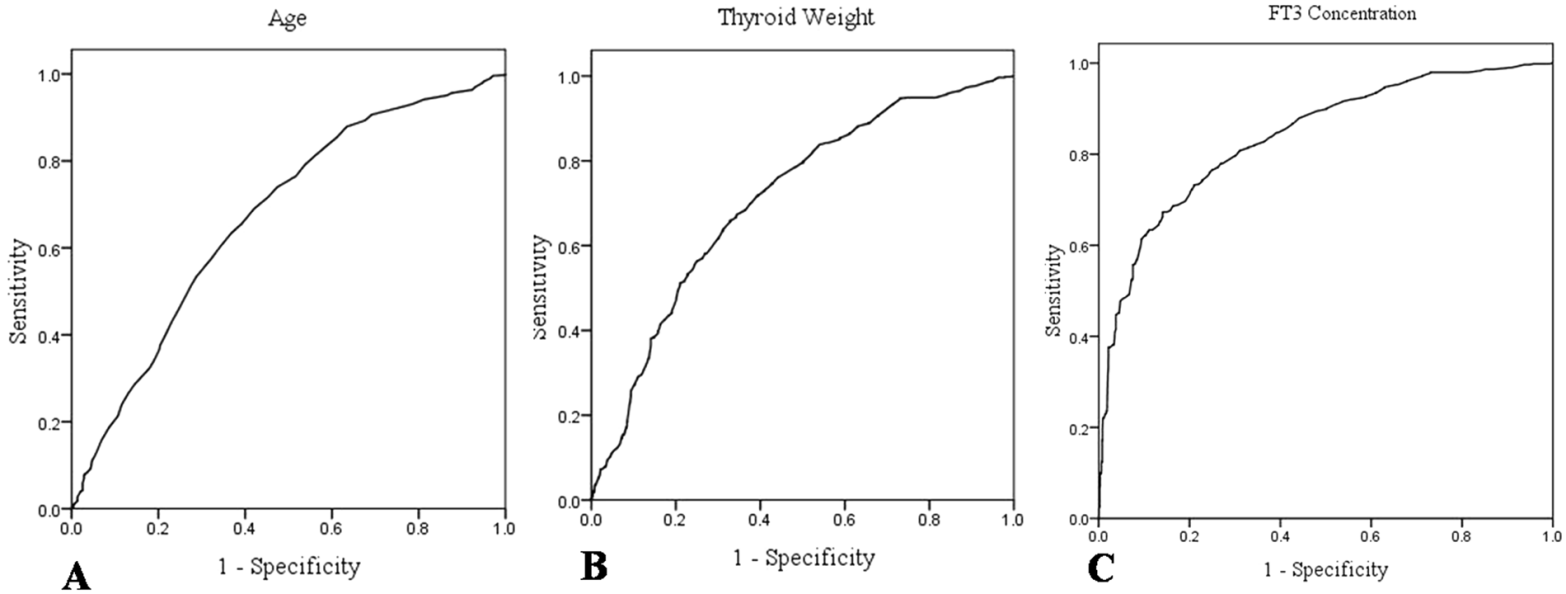
ROC curves for age (**A**), thyroid weight (**B**) and FT_3_ concentration (**C**) in predicting rapid thyroidal ^131^I turnover in GD patients.

Receiver operating characteristic (ROC) curves were drawn to evaluate the accuracy of age, FT_3_ concentration and thyroid weight in predicting rapid thyroidal ^131^I turnover (Fig. 1).

The optimal cutoffs were the values yielding maximum sums of sensitivity and specificity from the ROC curves^17, 18^. The results demonstrated that the optimal cutoff values for age and FT_3_ concentration were 38 years old and 35 pmol/l, at which the sensitivity and specificity were 63.3%, 63.2% (for age) and 75.3%, 73.1% (for FT_3_), respectively (area under curve [AUC]: 0.672; 95% CI: 0.646–0.697 and AUC: 0.815; 95% CI: 0.796–0.834, *P* < 0.001, respectively). Similarly, we found a thyroid weight threshold of 56 g, with a sensitivity of 67.3% and specificity of 65.6% for rapid ^131^I turnover (AUC: 0.710; 95% CI: 0.687–0.733, *P* < 0.001).

### Logistic regression analysis

Table 3 shows a multivariate logistic regression analysis of the potential risk factors of rapid ^131^I turnover. Variables that were significant in the univariate analysis were entered into the stepwise method. The multivariate logistic regression analysis revealed that patients with thyroid weight ≥56 g and FT_3_ concentration ≥35 pmol/l demonstrated a 3.7-fold and 7.6-fold higher probability of rapid ^131^I turnover, respectively, and cases with age < 38 years old showed a 2.3-fold higher probability. Additionally, female patients had a 2.2-fold higher probability of rapid ^131^I turnover.

**Table 3:** Comparison of predictors for rapid ^131^I turnover by multivariate logistic regression analysis.

Characteristics	OR	95% CI	*P* value
Gender (female vs. male)	2.214	1.757–2.791	<0.0001
Age (<38yrs vs. ≥38yrs)	2.333	1.906–2.856	<0.0001
thyroid weight (≥56 g vs. <56 g)	3.718	3.032–4.559	<0.0001
FT_3_ (≥35 pmol/l vs. <35 pmol/l)	7.625	5.857–8.563	<0.0001

FT_3_ = free triiodothyronine, FT_4_ = free thyroxine, OR = odds ratio, CI = confidence interval.

### Comparison of rapid ^131^I turnover in patients with thyroid weight <56 g or ≥56 g, age <38years or ≥38years and FT_3_ concentration <35 pmol/L or ≥35 pmol/L

**Figure 2:**
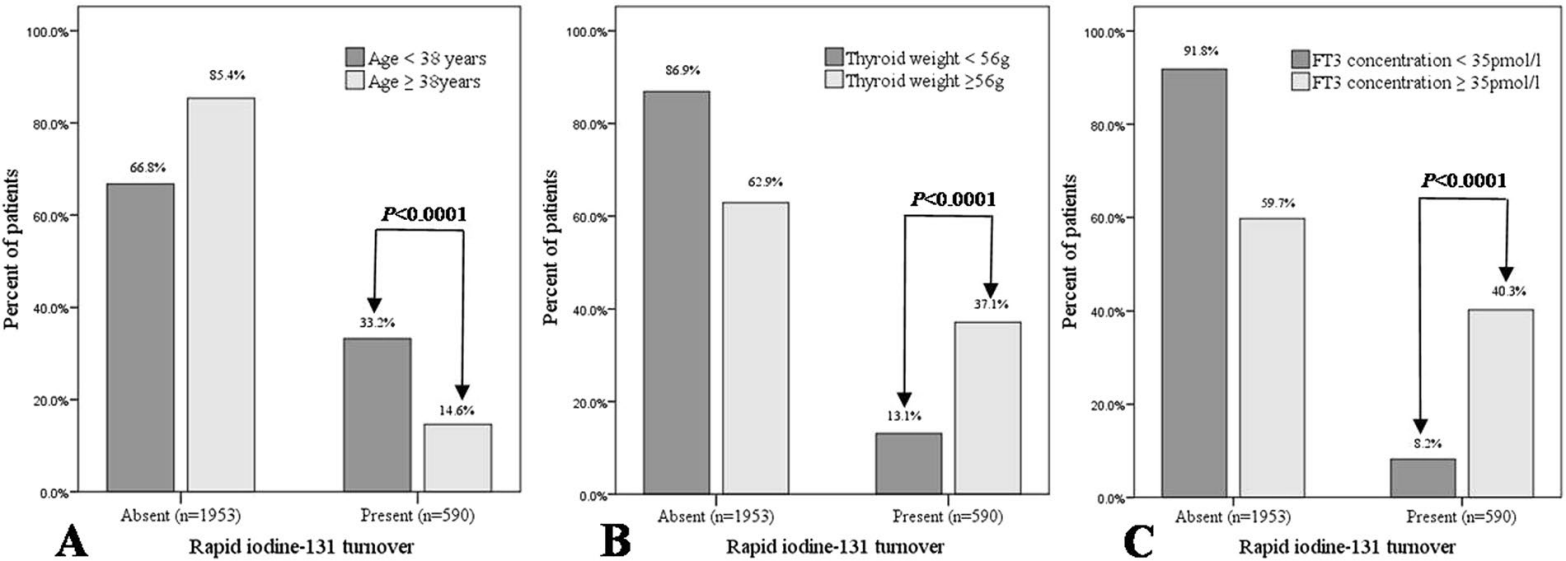
A comparative analysis of the percent of rapid ^131^I turnover between the patients with age <38 years or ≥38years (**A**), thyroid weight <56 g or ≥56 g (**B**) and FT_3_ concentration <35 pmol/l or ≥35 pmol/l (**C**).

A comparative analysis of the percent of rapid ^131^I turnover, using the chi square test, between the patients with thyroid weight <56 g or ≥56 g, age <38 years or ≥38years and FT_3_ concentration <35 pmol/l or ≥35 pmol/l was performed (Fig. 2).

We found a rapid ^131^I turnover rate of 37.1% among patients with thyroid weight ≥56 g and 13.1% with thyroid weight <56 g (*P* < 0.0001). Additionally, the rapid ^131^I turnover rates in patients with age <38 years and ≥38years were 33.2% and 14.6%, respectively *(P* < 0.0001). Similarly, we also found a rapid ^131^I turnover rate of 40.3% among patients with FT_3_ concentration ≥35 pmol/l and 8.2% with FT_3_ concentration <35 pmol/l (*P* < 0.0001).

## Discussion

Radioactive iodine (^131^I) therapy is the most common modality for treatment of hyperthyroidism in the United States^2^. About 80–95% of GD patients could be controlled after first dose of 131I therapy, which is a relatively safe, simple and effective form of therapy^4, 7, 9^. In our institution, only 195 patients (7.7%) remained hyperthyroid after first-dose RIT.

Rapid ^131^I turnover in the thyroid gland is an important feature of GD and can be observed in 12–32% of patients with GD^12–14^. In the present study, we reviewed a large-sample GD patients and found the prevalence of rapid ^131^I turnover is 23.2% in our patient population. Some investigators reported that rapid ^131^I turnover was a strong predictor of RIT failure^12, 14, 15^, and Aktay *et al*.^12^ found up to 55% of the GD patients with rapid ^131^I turnover failed to respond to the initial ^131^I therapy. Similarly, our study showed patients with ^131^I uptake ratio of ≥1 have a higher first-dose RIT failure rate when comparing against those with ^131^I uptake ratio of <1 (12.0% vs. 6.3%, *P* < 0.0001), although we delivered a higher concentration of ^131^I per gram of thyroid tissue to patients with rapid ^131^I turnover in our routine work. The higher failure rate of ^131^I therapy among patients with rapid ^131^I turnover might be explained by the rapid clearance or turnover of iodine-131 from the thyroid gland, which results in a shorter effective half-life of ^131^I with less radiation subsequently delivered to the gland^12^. Therefore, patients with rapid ^131^I turnover should receive a larger dose of ^131^I in order to obtain higher RIT success rates.

The relatively high rapid ^131^I turnover and first-dose RIT failure rate in patients with GD highlight the importance of identifying predictors of rapid ^131^I turnover in this patient population. In our study, no differences were found in the disease duration, 4 h or 24 h thyroid ^131^I uptake, and complications. Meanwhile, gender, age, FT_3_/FT_4_ concentration, antithyroid medication, time of ATD, thyroid textures and thyroid weight could be used as the potential variables to predict rapid ^131^I turnover in the univariate analysis. Female patients had a higher rapid ^131^I turnover rate than males (26.5% vs. 15.9%). The FT_3_ and FT_4_ concentrations were higher in cases with rapid ^131^I turnover (38.2 pmol/l vs. 31.3 pmol/l, and 86.5 pmol/l vs.78.9 pmol/l, respectively). The values of thyroid weight in patients with ^131^I uptake ratio of ≥1 were heavier than those with ^131^I uptake ratio of <1 (68.0 g vs. 48.5 g), and patients with rapid ^131^I turnover were younger (33yrs vs. 42yrs). Furthermore, using multivariate logistic analysis, we found that gender, FT_3_ concentration, thyroid weight and age were the independent factors related to rapid ^131^I turnover. In our patient population, we verified that female patients had a 2.2-fold higher probability of rapid ^131^I turnover. Moreover, patients with thyroid weight ≥56 g and FT_3_ concentration ≥35pmol/l had a 3.7-fold and 7.6-fold higher probability of rapid ^131^I turnover, with an accuracy of 71.0% and 81.5%, respectively. Additionally, we verified that patients with age <38 years old showed 2.3 times more risk of rapid ^131^I turnover (accuracy 67.2%).

Rapid ^131^I turnover has been ascribed to the so-called “small iodine pool syndrome,” which can be seen in patients pretreated with ATD^19, 20^. Although ATD have short half-lives in blood, there is a high concentration and retention in the intra-thyroid environment, which may lead to a reduction in ^131^I uptake and effective half-life of ^131^I in the thyroid^19^. Thus, the ^131^I turnover is faster in comparison to patients who were not treated. Additionally, patients who maintained anti-thyroid drug use during RIT also had a 4.9-fold higher risk of treatment failure in comparison to those who discontinued the medication^21^. In our study, 74.9% patients with rapid ^131^I turnover had received antithyroid medications, comparing to 66.6% without rapid ^131^I turnover (*P* < 0.001). However, a significant different finding in the univariate analysis was not upheld in the multivariate model, indicating that antithyroid medication prior to RIT is not considered to be a significant factor predicting rapid ^131^I turnover.

As with most retrospective studies, this study has certain shortcomings. Firstly, the main weakness is the lack of data on thyroid autoantibodies titers, especially anti-thyrotrophin receptor antibody (TRAb) level which could be helpful in predicting disease severity and chance of RIT failure, whereas it was not available as a routine laboratory assessment during the time of data collection and we were unable to include it in our statistical analysis. Secondly, in this study, we only defined multiple ^131^I therapies as first-dose RIT failure, however, although few, some patients lost to follow-up or chose other forms of treatment such as antithyroid medication or surgery after the initial RIT. Therefore, first-dose failure rate in this study was slightly lower.

In conclusion, the 4- to 24-hour ^131^I uptake ratio appears to be a practical index for predicting early peaking of ^131^I uptake in GD patients. The incidence of rapid ^131^I turnover was high, which was expected in patients presenting larger goiters, younger age, higher FT_3_ concentration and females, particularly those with thyroid weight ≥56 g, age <38 years, FT_3_ concentration ≥35 pmol/l.

## Materials and Methods

### Subjects

Between June 2007 and March 2014, the medical records of hyperthyroid patients consecutively referred to the Thyroid Clinic for ^131^I therapy were reviewed. The ^131^I dose (MBq) = ^131^I dose for per gram of thyroid tissue (MBq/g) × thyroid weight (g)/24h-RAIU. Of all the 2940 patients, a total of 2543 patients (793 men and 1750 women; age, 10–80 years) with the clinical diagnosis of GD were selected and 350 cases with other etiologies for hyperthyroidism, including multinodular goiter, plummer’s disease and hashimoto’s thyroditis were excluded. Additionally, the remaining 47 patients who had received RIT before were also excluded. GD was diagnosed on the basis of diffuse goiter, elevated 4- or 24-hour RAIU of the thyroid gland, thyrotoxicosis, and/or positive TRAb. All medications that could interfere with thyroidal ^131^I uptake, such as seafood and some drugs (methimazole, propylthiouracil, compound iodine solution, probanthine, and so on), were stopped at least one week before RAIU measurements.

This study was approved by the medical ethics research committee of Tianjin Medical University General Hospital and written informed consent was obtained from each patient. We confrmed that all methods were carried out in accordance with the relevant guidelines and regulations.

### Data collection and grouping

Data on gender, age, disease duration, thyroid function tests, with or without ATD, time of ATD, thyroid weight, 4 h/24 h thyroid ^131^I uptake, thyroid textures (soft, moderate or stiff), thyroid nodules or not, with complications or not prior to RIT were collected for all patients.

All the patients were divided into 2 groups depending on present or absent with rapid thyroidal ^131^I turnover (early peaking of ^131^I uptake), which was defined as an early (approximately 4 hour)/late (approximately 24 hour) ^131^I uptake ratio of ≥1.

### RAIU, thyroid function tests and thyroid weight

The RAIU value was obtained at 4 and 24 hour after an oral tracer dose (about 74 kBq) of ^131^I through a nuclear multifunctional instrument/counter (MN-6300XT Apparatus, Technological University, China). The thyroidal ^131^I uptake was calculated according to the following equation: RAIU (%) = (neck counts − background counts) × 100/(standard counts − background counts). Thyroid function tests, including serum FT_3_ (normal range: 3.1–6.8 pmol/L) and FT_4_ (normal range: 12–22 pmol/L) concentrations etc, were measured by chemiluminescent immunoassays (Cobas 6000, Roche Diagnostics GmbH, Mannheim, Germany). Length, breadth, and depth of each lobe was measured respectively, the volume of each lobe was calculated using the formula for a prolate ellipsoid, and estimated thyroid weight(g) = length × breadth × depth × π/6.^22^

### Statistical analysis

Statistical analysis was performed using SPSS (Statistical Package for Social Sciences) 12.0 for windows (SPSS, Chicago, IL, USA). Continuous and categorical variables were expressed as mean ± standard deviation (SD) (median [range]) and count (percentage), respectively. A chi square test was used to verify association or compare proportions. To compare continuous variables in the 2 groups, the Mann-Whitney U test was performed due to non-normal distributions. ROC curves were plotted to identify the best threshold for the potential predictors of rapid ^131^I turnover. AUC was used as an estimation of diagnostic accuracy. To identify associated factors of rapid ^131^I turnover, we performed multivariate logistic regression analysis with a variable entrance criterion of 0.05 or less. All *P* values presented were two-tailed, and values <0.05 were considered to be statistically significant.
